# LL-37 Induces Polymerization and Bundling of Actin and Affects Actin Structure

**DOI:** 10.1371/journal.pone.0050078

**Published:** 2012-11-26

**Authors:** Asaf Sol, Edna Blotnick, Gilad Bachrach, Andras Muhlrad

**Affiliations:** 1 Institute of Dental Sciences, The Hebrew University-Hadassah School of Dental Medicine, The Hebrew University of Jerusalem, Jerusalem, Israel; 2 Department of Medical Neurobiology, The Institute for Medical Research-Israel-Canada, The Hebrew University of Jerusalem, Jerusalem, Israel; University of Muenster, Germany

## Abstract

Actin exists as a monomer (G-actin) which can be polymerized to filaments) F-actin) that under the influence of actin-binding proteins and polycations bundle and contribute to the formation of the cytoskeleton. Bundled actin from lysed cells increases the viscosity of sputum in lungs of cystic fibrosis patients. The human host defense peptide LL-37 was previously shown to induce actin bundling and was thus hypothesized to contribute to the pathogenicity of this disease. In this work, interactions between actin and the cationic LL-37 were studied by optical, proteolytic and surface plasmon resonance methods and compared to those obtained with scrambled LL-37 and with the cationic protein lysozyme. We show that LL-37 binds strongly to CaATP-G-actin while scrambled LL-37 does not. While LL-37, at superstoichiometric LL-37/actin concentrations polymerizes MgATP-G-actin, at lower non-polymerizing concentrations LL-37 inhibits actin polymerization by MgCl_2_ or NaCl. LL-37 bundles Mg-F-actin filaments both at low and physiological ionic strength when in equimolar or higher concentrations than those of actin. The LL-37 induced bundles are significantly less sensitive to increase in ionic strength than those induced by scrambled LL-37 and lysozyme. LL-37 in concentrations lower than those needed for actin polymerization or bundling, accelerates cleavage of both monomer and polymer actin by subtilisin. Our results indicate that the LL-37-actin interaction is partially electrostatic and partially hydrophobic and that a specific actin binding sequence in the peptide is responsible for the hydrophobic interaction. LL-37-induced bundles, which may contribute to the accumulation of sputum in cystic fibrosis, are dissociated very efficiently by DNase-1 and also by cofilin.

## Introduction

Actin is the most abundant protein in the eukaryotic cell. It is a highly dynamic structural protein with multiple functions in cell physiology which include cytoskeleton formation, cell division, motility, adhesion, signaling and more [1]. Actin exists in either monomer- globular (G) or polymer- filament (F) form. These forms can interconvert into each other by a plethora of different factors. G-actin, which is negatively charged, is polymerized into F-actin by an increase in monovalent or divalent cation concentration and by positively charged proteins and peptides.

F-actin filaments may form bundles via specific actin bundling proteins [2], which cross-link two filaments when their two discrete actin-binding sites attach to separate filaments. These proteins, including α-actinin, filamin, fimbrin, spectrin and others, form tightly packed, well organized bundles containing parallel arranged filaments [3], [4]. The other group of actin bundling factors are the polycations, including polycationic proteins or peptides and polyamines. This group induces bundle formation via non-specific electrostatic interactions by eliminating repulsion between negatively charged actin filaments [5]. LL-37 [6], lysozyme [7] MARCKS [8], [9], ENA/VASP [10], fesselin [11], [12] and calponin [13], [14] all belong to this group. Polycationic polyamines such as spermine and spermidine [15], [16] and polylysine [17] also bundle F-actin. Site specific hydrophobic interactions between actin and polycation might contribute to the bundling especially at high ionic strength, where the bundling effect of polycations decreases because the relatively high concentration of monovalent cations masks the electrostatic interactions between polycations and actin [18]. The bundling process of F-actin by lysozyme, spermine and polylysine was recently studied in some detail [19], [20].

LL-37-induced actin polymerization and bundling was chosen as the subject of this study. LL-37 is a 37 amino acid polycationic peptide with 6 net positive charges and is the only known member in human of the cathelicidin family of host defense peptides [21]. In addition to its antimicrobial activity, the host defense activities of LL-37 include chemoattraction [22], cytokine release [23], tissue regeneration [24], inhibition of immunostimulation [25] and apoptosis 21,26. LL-37 is actively taken up by lung epithelial cells [27]. There, it increases the F-actin content in the cytoskeleton, and acts to enhance cell stiffness and decrease the membrane permeability of these cells [28] **.** LL-37 has been shown to bundle F-actin filaments [6]. The LL-37-induced actin bundles contribute to the accumulation of sputum, a viscous mucus, in the airways of cystic fibrosis patients and a primary cause of bacterial infections and death in this disease [29]. The antimicrobial activity of LL-37 attached to actin bundles was reported to be significantly reduced [6]. Gelsolin and polyanions dissolve actin bundles [30] and restore LL-37′s antibacterial activity [6]. Despite these significant advances, important processes, such as polymerization of G-actin by LL-37, kinetics of bundle formation, characterization of actin-LL-37 interaction at molecular level and the reaction of LL-37-induced bundles with actin binding and severing proteins remain uncharacterized and poorly understood.

In this study, we examined the LL-37-induced polymerization of MgATP-G-actin and the bundling of Mg-F-actin, and compared them to the well characterized polymerization and bundling of actin by the antibacterial polycationic protein lysozyme [20]. We show that LL-37 polymerizes actin only at concentrations greater than twice that of actin. Low concentrations of LL-37, which are not sufficient to polymerize actin, inhibit actin polymerization induced by MgCl_2_ or NaCl. LL-37-induced F-actin bundles are less sensitive to ionic strength when compared to sLL-37 (scrambled LL-37) or lysozyme induced bundles. Substoichiometric LL-37 concentrations relative to actin accelerate the subtilisin digestion of the protein. Our results indicate that in addition to electrostatic interactions there are specific hydrophobic interactions between LL-37 and actin. These interactions may involve the DNase1 binding (D) loop since DNase1 dissociates LL-37 induced F-actin bundles very efficiently.

## Materials and Methods

### Materials

N-(1-pyrene) maleimide was obtained from Molecular Probes (Eugene, OR). Hen lysozyme, DNase1, ATP, ADP, dithiotreitol (DTT), and EGTA were purchased from Sigma Chemical Co. (St Louis, MO). LL-37 (LLGDFFRKSKEKIGKEFKRIVQRIKDFLRNLVPRTES) peptide and scrambled LL-37 (sLL-37) peptide (GLKLRFEFSKIKGEFLKTPEVRFRDIKLKDNRISVQR) were purchased from (Genemed Synthesis Inc., San Antonio,TX ). The peptides were purified by HPLC and purity (greater than 90%) was determined by Mass Spectrometry. Yeast cofilin was a generous gift from Prof. Emil Reisler Dept. of Chemistry and Biochemistry, Univ. of California Los Angeles CA.

### Preparation of Actin

CaATP-G-actin was prepared from acetone dried powder derived from the back and leg muscles of rabbit by the method of Spudich and Watt [31] that even without gel filtration yields highly homogeneous actin in purity greater than 90%. CaATP-G-actin was stored in a buffer containing 5 mM TrisHCl, 0.2 mM CaCl_2_, 0.2 mM ATP, 0.5 mM β-mercaptoethanol, pH 8.0 (CaATP-G-buffer). MgATP-G-actin was obtained by incubating CaATP-G-actin with 0.2 mM EGTA and 0.1 mM MgCl_2_ at room temperature for 5 min. MgATP-G-actin was diluted for further treatments in MgATP-G-buffer containing 5 mM MOPS, 0.1 mM MgCl_2_, 0.2 mM EGTA, 0.2 mM ATP and 0.5 mM DTT, pH 7.4. MgF-actin was polymerized from MgATP-G-actin or CaATP-G-actin by 30 min incubation with 2 mM MgCl_2_ at room temperature. The concentration of unlabeled rabbit skeletal muscle α-G-actin was determined spectrophotometrically using the extinction coefficients E^1%^_290_ = 11.5 cm^−1^. (The optical density of actin was measured in the presence of 0.5 M NaOH, which shifts the maximum of absorbance from 280 nm to 290 nm). Molecular masses of skeletal actin, yeast cofilin, hen-lysozyme and LL-37 were assumed to be 42 kDa, 15.9 kDa, 14.3 kDa and 4.5 kDa respectively.

### Pyrene Labeling

Labeling of Mg-F-actin at Cys-374 with pyrene maleimide was carried out according to Kouyama and Mihashi [32] with some modifications. CaATP-G-actin was filtered through a PD-10 column (GE Healthcare) equilibrated with β-mercaptoethanol-free CaATP-G-buffer. After filtration, actin (1 mg/ml) was polymerized by 2 mM MgCl_2_ and 100 mM KCl at room temperature for 30 min, and reacted with pyrene maleimide (16 µg/ml) on ice, for one hour. The reaction was terminated with 1 mM DTT. Labeled F-actin was centrifuged at 129,151×*g* for two hours, the pellet was resuspended in Ca-ATP-G-buffer and depolymerized by dialyzing in this buffer for over 36 hours at 4°C. Finally, actin was centrifuged again at 129,151×*g* for two hours. The supernatant contained the purified pyrene-labeled CaATP-G-actin. The concentration of modified actin was determined by the procedure of Bradford [33] using unmodified actin as a standard. The extent of labeling, which was measured by using pyrene extinction coefficient E_344 nm_ = 22000 cm^−1^M^−1^, was ∼100%.

### Fluorescence and Light Scattering Measurements

Actin polymerization was followed as an increase in fluorescence of pyrene-labeled G-actin [32], which was added to unlabeled G-actin in 10–15%. The time course of pyrene-labeled actin polymerization was monitored by measuring fluorescence increase (with 365 nm excitation and 386 nm emission wavelengths) in a PTI spectrofluorometer (Photon Technology Industries, South Brunswick, NJ). Bundling of MgF-actin was followed by increase in light scattering [20]. The time course of light scattering changes was also measured in a PTI spectrofluorometer, with both excitation and emission wavelengths adjusted to 450 nm. All fluorescence and light scattering measurements were carried out at 22°C.

### Monitoring Bundling by Low Speed Sedimentation

After addition of polycations, actin samples were centrifuged at 20,800×*g* at 4°C for 8 min (in an Eppendorf centrifuge). The supernatants were run using SDS-PAGE and analyzed by densitometry using the TINA 2.07d software.

### Binding Measurement of LL-37 or Scrambled LL-37 to G-actin by Surface Plasmon Resonance

The preparation of the sensor surfaces and the interpretation of the sensorgrams were performed using BIACORE 3000 system (GE). For the immobilization procedure, a CM5 chip (GE) was activated using amine coupling kit (GE) by the standard protocol. The running buffer used was 10 mM HEPES buffer pH 7.4, 150 mM NaCl, and 0.005% tween 20. The flow rate during immobilization was 5 µl/min. Following activation, actin (10 µg/ml) was injected to give a final level of actin binding of 1500–1800 RU (Resonance Units). The remaining activated groups were blocked by injection of 1 M ethanolamine, pH 8.5. All experiments were conducted at 25°C using a flow rate of 40 µl/min. Series of LL-37 concentrations (up to 500 nM) were injected. Each injection was followed by regeneration using 10 µl of 10 mM NaOH. Evaluation was done using BIAevaluation software (version 4.1). Affinity constant was calculated by using the 1∶1 Langmuier model. For sLL-37 a steady state affinity model was used due to fast equilibration. Binding was measured as Resonance Units (RU)/s. The SPR signal obtained in each individual reaction cycle was recorded as a sensorgram, which is a real-time pattern plotted in RU versus time (s). The KD. Experiments were repeated three times.

## Results

### Polymerization of MgATP-G-actin by LL-37

**Figure 1:**
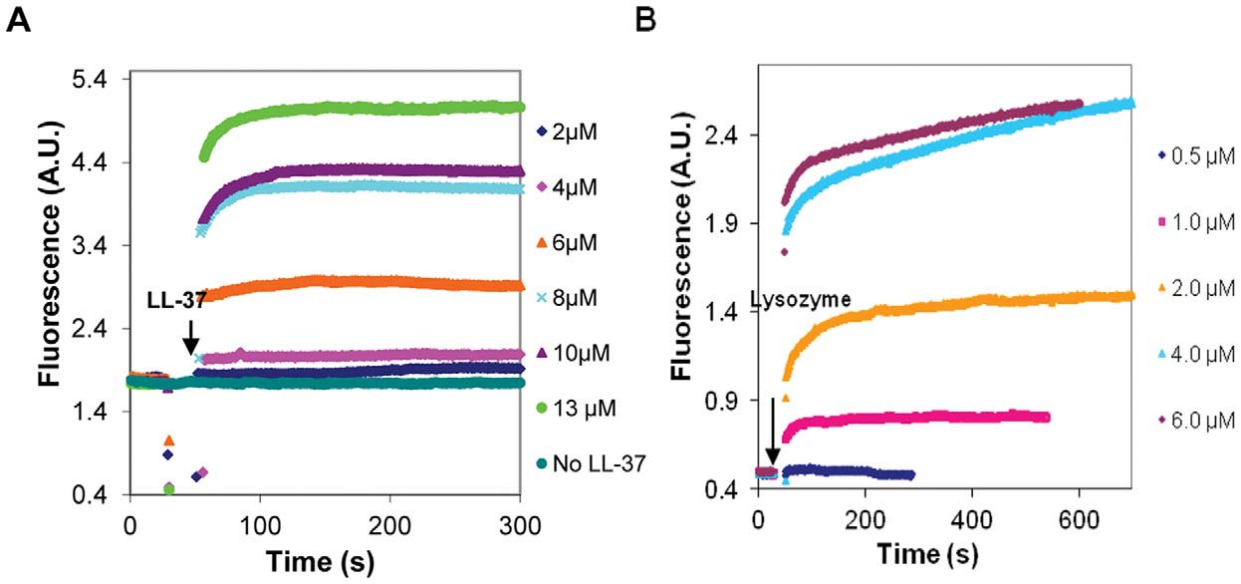
Polymerization of MgATP-G-actin by LL-37 and lysozyme at low ionic strength MgATP-G-buffer. LL-37 (A), or lysozyme (B) were added to pyrene labeled (10% labeling ratio) 2 µM MgATP-G-actin and the polymerization was followed by increase in pyrene fluorescence. Presented data are representative of three independent experiments.

**Figure 2:**
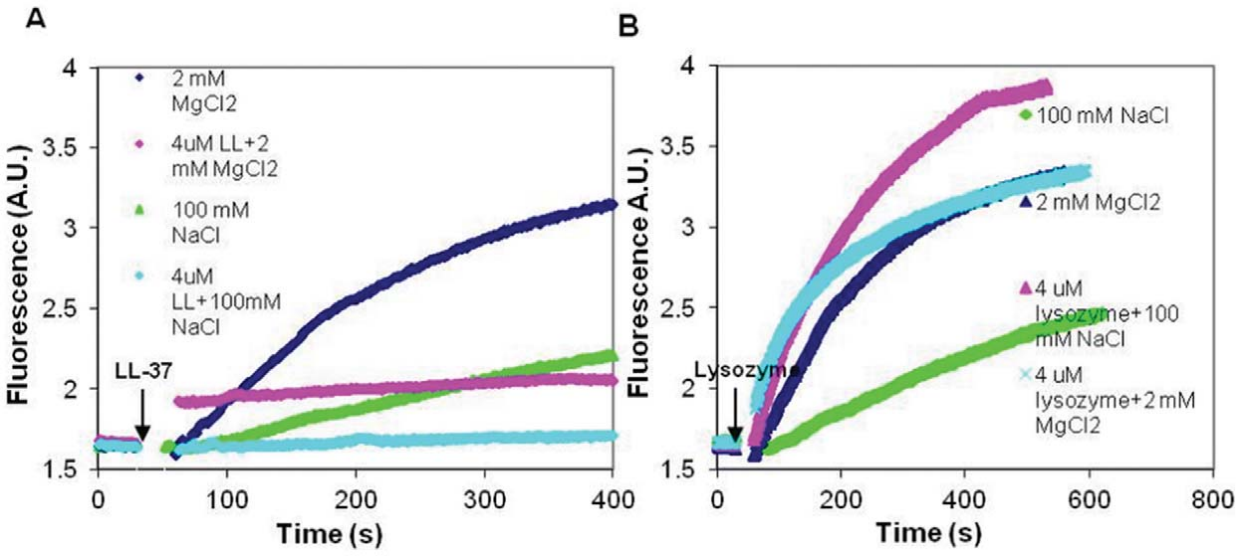
Effect of LL-37and lysozyme, on the polymerization of MgATP-G-actin induced by 100 mM NaCl or 2 mM MgCl_2_. 4 µM LL-37 (A) or lysozyme (B) were added (simultaneously with NaCl or MgCl_2_) to pyrene labeled (10% labeling ratio) 2 µM MgATP-G-actin and the polymerization was followed by an increase in pyrene fluorescence. Presented data are representative of three independent experiments.

In low ionic strength MgATP-G-buffer, LL-37 was found to polymerize MgATP-G-actin (Figure 1A). The onset of polymerization was fast and its extent was dependent on the concentration of LL-37. Significant polymerization was observed when the concentration of LL-37 was three times higher than that of MgATP-G-actin (6 µM LL-37, and 2 µM actin) (Figure 1A). Lysozyme polymerized MgATP-G-actin at half of the concentration of MgATP-G-actin (1 µM lysozyme and 2 µM actin) (Figure 1B). One may suppose that lysozyme polymerizes G-actin at lower concentrations than LL-37; because its net positive charge [34] is higher than that of LL-37 [6]. However, LL-37’s affinity to MgATP-G-actin also seems to be high as implied by the finding that LL-37 affects G-actin even when its concentration is too low to induce polymerization. Under these conditions, LL-37 inhibits polymerization induced by 2 mM MgCl_2_ or 100 mM NaCl (Figure 2A) while similar concentrations of lysozyme have no inhibitory effect (Figure 2B). These results may indicate that monomeric actin forms complexes with LL-37 that at low LL-37 concentrations inhibit salt-induced actin polymerization.

### Bundling of MgF-actin by LL-37

**Figure 3:**
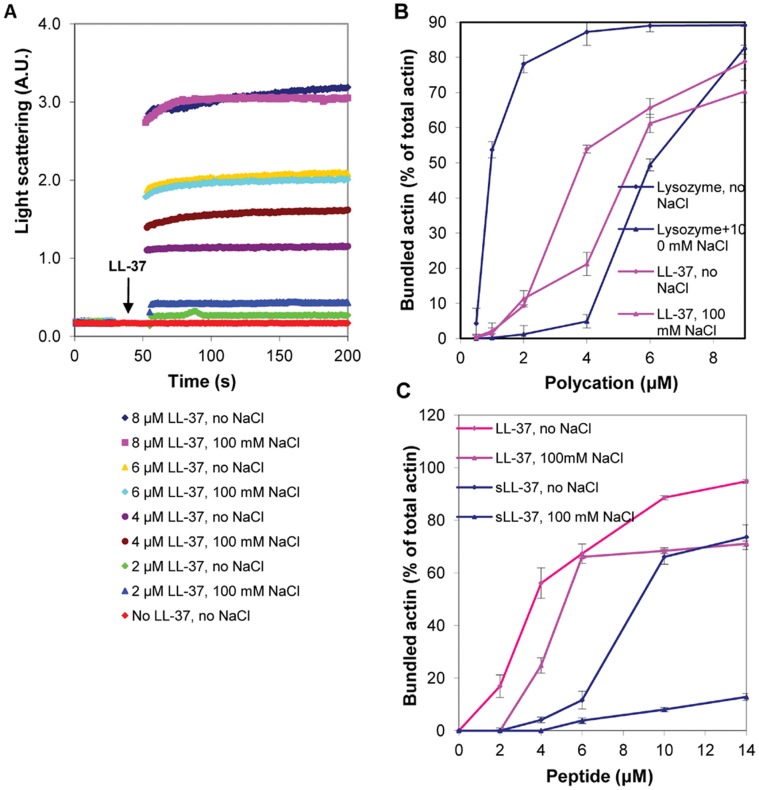
LL-37, scrambled LL-37 and lysozyme induced bundle formation of Mg-F-actin at low and physiological (100 mM NaCl) ionic strength. (**A**) Kinetics of LL-37-induced bundle formation: LL-37 (2–8 µM) was added to MgF-actin (2 µM) and bundle formation was followed as an increase in light scattering at 350 nm. Presented data are representative of three independent experiments. (**B**) Extent of LL-37 and lysozyme induced bundle formation: LL-37 or lysozyme (0.5–9 µM) were added to Mg-F-actin (4 µM). Samples were centrifuged at 20,800×g, for 8 min and the supernatants were analyzed by SDS-PAGE and densitometry. (**C**) Extent of LL-37 and scrambled LL-37 induced bundle formation: LL-37 or scrambled LL-37 (2–14 µM) were added to MgF-actin (4 µM) and bundle formation was measured as in (B). The presented data are mean and standard deviation of three independent experiments in (B) and (C).

Bundling kinetics of MgF-actin by LL-37, lysozyme and scrambled LL-37 were studied by increase in light scattering and the extent of bundling was determined using low speed centrifugation. The bundling of MgF-actin by LL-37 is also shown by electron microscopy (Figure S1). Mg-F-actin bundle formation was very fast both with LL-37 (Figure 3A) and lysozyme [20]. The extent of bundling was dependent on the concentration and the nature of the polycation used (Figure 3B). At low ionic strength more than 50% of Mg-F-actin was bundled upon addition of lysozyme (1 µM) to Mg-F-actin (4 µM). A similar degree of bundling was observed with LL-37 when its concentration was equal to that of actin (4 µM) (Figure 3B). At low ionic strength scrambled LL-37 induced significant bundling when its concentration was 2.5 fold higher than that of MgF-actin (Figure 3C). These results are similar to those obtained for MgATP-G-actin polymerization by lysozyme and LL-37. Bundling of F-actin by polycations is ionic-strength-sensitive and bundles induced by polycations dissociate (unbundle) at physiological ionic strength [20]. This is also observed with lysozyme where a six fold higher concentration of lysozyme (6 µM ) was needed for half maximal bundling of 4 µM Mg-F-actin at physiological (100 mM NaCl) than at low ionic strength (1 µM lysozyme) (Figure 3B). With scrambled LL-37, whose net number of positive charges are identical to those of LL-37, even 14 µM were insufficient to cause significant bundling in the presence of 100 mM NaCl, while at low ionic strength more than 60 percent of MgF-actin bundled at 10 µM concentration of sLL-37 (Figure 3C). The results show the competing effect of ionic strength and polycation concentration on bundling. Unlike with lysozyme and sLL-37, the kinetics (Figure 3A) and the extent (Figure 3B and C) of bundling by LL-37 are practically identical at low and at physiological ionic strength. The decreased salt sensitivity of LL-37-induced bundles may indicate that the interactions between LL-37 and actin are partially electrostatic and partially hydrophobic.

**Figure 4:**
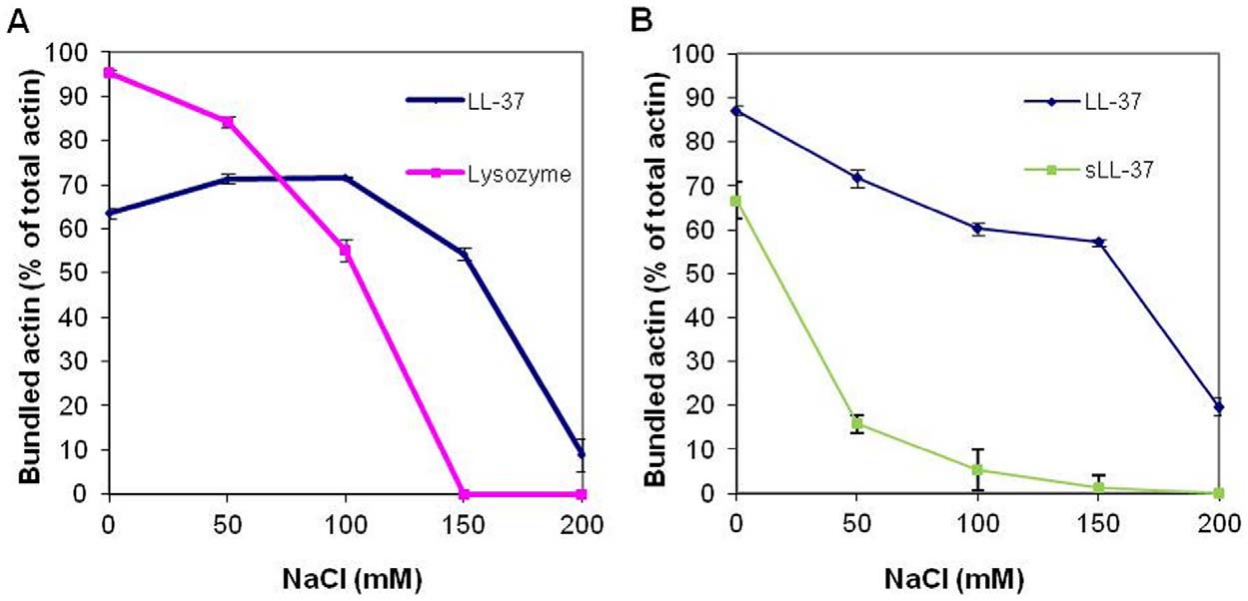
NaCl concentration dependent bundling of MgF-actin by LL-37, lysozyme and sLL-37. (**A**) 6 µM lysozyme or LL-37 were added to Mg-F-actin (4 µM). (**B**) 10 µM LL-37 or sLL-37 were added to 4 µM MgF-actin After 10 min incubation at room temperature samples were centrifuged at 20,800×g for 8 min. The supernatants were analyzed by SDS-PAGE and densitometry. The presented data are mean and standard deviation of three independent experiments.

We studied the effect of NaCl concentration on the stability of bundles formed from 4 µM MgF-actin, that were induced by 6 µM LL-37 or lysozyme (Figure 4a), or by 10 µM LL-37 or sLL-37 (Figure 4B). The LL-37-induced bundles were reasonably stable in the 0–150 mM NaCl range and dissociated only at 200 mM NaCl. In contrast, the stability of lysozyme and sLL-37 induced bundles was decreased already at 50 mM and the bundles completely dissociated in 150 mM NaCl. These results support our assumption that reduced salt sensitivity of the LL-37-induced bundles is due to hydrophobic interactions between LL-37 and actin.

### Effect of LL-37 on Subtilisin Digestion of CaATP-G-, MgATP-G-actin and Mg-F-actin

**Figure 5:**
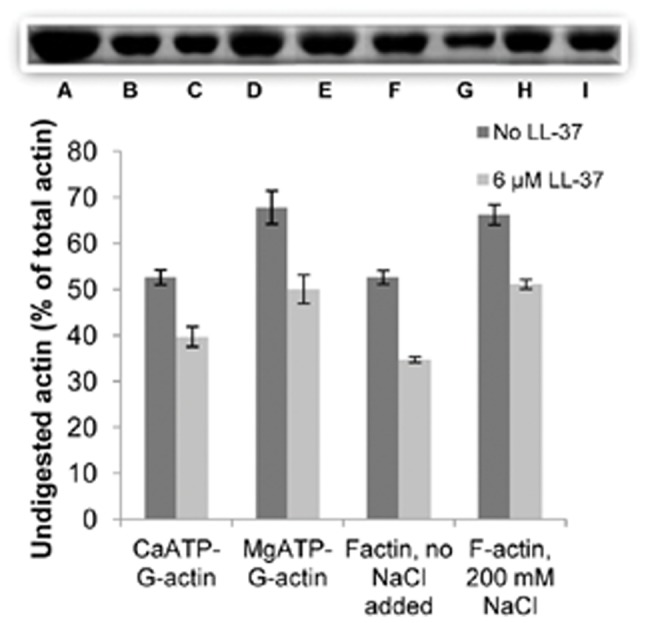
Effect of 6 **µM LL-37 on subtilisin digestion of CaATP-G-actin, MgATP-G-actin and Mg-F-actin.** 8 µM CaATP-G-actin or 8 µM MgATP-G-actin were digested by 4 µg/ml subtilisin for 2 min, MgF-actin (8 µM) was digested by 20 µg/ml subtilisin for 30 min in the presence or absence of 6 µM LL-37. Digestions were carried out at 22°C and quenched by 1 mM PMSF. Samples were analyzed by 12% SDS-PAGE and densitometry. Gel insert shows representative actin bands: (A), actin only; (B), CaATP-G-actin, subtilisin, no LL-37; (C), CaATP-G-actin,subtilisin and LL-37, (D) MgATP-G-actin, subtilisin, no LL-37, (E) MgATP-G-actin, subtilisin and LL-37, (F) Mg-F-actin, subtilisin, no LL-37, (G) Mg-F-actin, subtilisin and LL-37, (H) Mg-F-actin, 200 mM NaCl, subtilisin, no LL-37, (I) Mg-F-actin, 200 mM NaCl, subtilisin and LL-37. The presented quantitation data are mean and standard deviation of three independent experiments.

Subtilisin at low concentrations cleaves G-actin between Met-47 and Gly-48 [35]. In order to cleave F-actin, about a magnitude higher concentration of subtilisin is needed. The subtilisin digestion of actin is dependent on the structure of the protein. For example, the exchange of tightly bound ATP to ADP in the nucleotide binding cleft, significantly decreases the rate of actin cleavage by subtilisin [36]. Addition of cofilin affects MgF-actin’s structure, and strongly accelerates the digestion of actin filaments by subtilisin [37]. In light of our assumption that the binding of LL-37 to actin is partially hydrophobic, and may affect actin structure, we examined the effect of LL-37 on actin digestion by subtilisin (Figure 5). CaATP- and MgATP-G-actin (8 µM) were digested by subtilisin (4 µg/ml) for 2 minutes. Mg-F-actin (8 µM) was digested in the presence and absence of 200 mM NaCl, by 20 µg/ml subtilisin for 30 min. Digestions were carried out in the presence or absence of 6 µM LL-37, which in all cases enhanced the subtilisin cleavage in a statistically significant manner (Figure 5). These results indicate that LL-37 binds both the monomer and polymer forms of actin. It also binds F-actin in the presence of 200 mM NaCl, which abolish electrostatic interactions between F-actin and LL-37 (Figure 4). Thus, the binding of LL-37 to actin is at least partially hydrophobic and induces changes in actin structure, which is manifested in increased sensitivity toward subtilisin.

### Surface Plasmon Resonance Measurements of Binding of LL-37 to Immobilized G-actin

**Figure 6:**
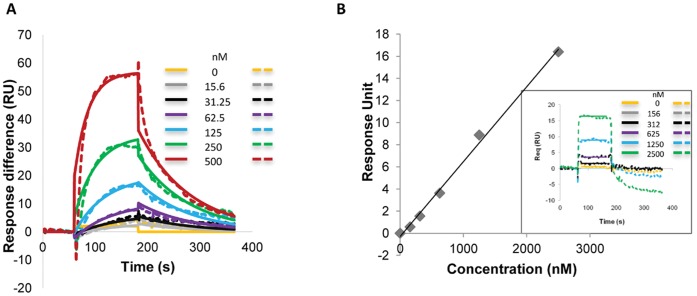
Binding measurements of LL-37 and of scrambled LL-37 to G-actin at high ionic strength. Biacore sensorgram [fit (solid) and experimental (dashed)] showing the interactions between LL-37 (A) or scrambled LL-37 (B) and actin, using series of concentrations of 0–500 nM (A), 0–2500 nM (B). Calculation for sLL-37 (B) were performed using a steady state affinity model based on the sensorgram shown as an insert in B. Presented data are representative of three independent experiments.

In light of the subtilisin digestion results, which showed that LL-37 binds G-actin and affects its structure, we measured the dissociation constants of LL-37 and of scrambled LL-37 from CaATP-G-actin using the SPR BIACORE approach (Figure 6). CaATP-G-actin was bound to the sensor chip at low ionic strength and washed. LL-37 or sLL-37 were applied to the surface-bound G-actin followed by washing with the 150 mM NaCl supplemented CaATP-G-buffer (G-actin was not polymerized by this high ionic strength buffer, because it was bound to the chip surface). The results presented in Figure 6A show that LL-37 binds strongly to G-actin (Table 1) in the presence of 150 mM NaCl, which masks most of the electrostatic interactions between actin and LL-37. The KD value of LL-37 binding obtained in this measurement is slightly different when compared with the results of the bundling experiments (Figure 3C). This might result from the fact that the binding of LL-37 was measured to monomeric CaATP-G-actin, while the bundling experiments were carried out with polymeric MgF-actin. sLL-37 binds very weakly to G-actin under the same conditions (Figure 6B, Table 1). These results indicate a strong, sequence dependent hydrophobic interaction between LL-37 and actin. It appears that LL-37 contains an actin binding sequence, which is perturbed by scrambling, that abolished the hydrophobic interaction.

**Table 1:** Dissociation constants of LL-37 bound to immobilized G-actin using the BIACORE 3000 system.

	KD (M)
LL-37	1.86×10^−7^
Scrambled LL-37	1.38×10^−4^

### Dissociation of LL-37-induced Mg-F-actin Bundles by DNase1 and Cofilin

Bundles of actin filaments are major contributors to the viscosity of sputum in the airways of cystic fibrosis patients. LL-37, which also has been found in the sputum, may contribute to actin bundle formation [38]. Gelsolin, an actin filament severing protein was found to significantly reduce the viscosity of sputum [30] and dissociate LL-37-induced actin filament bundles [38]. Here, we studied the effect of cofilin [39] and DNase1 on the stability of LL-37-induced bundles.

**Figure 7:**
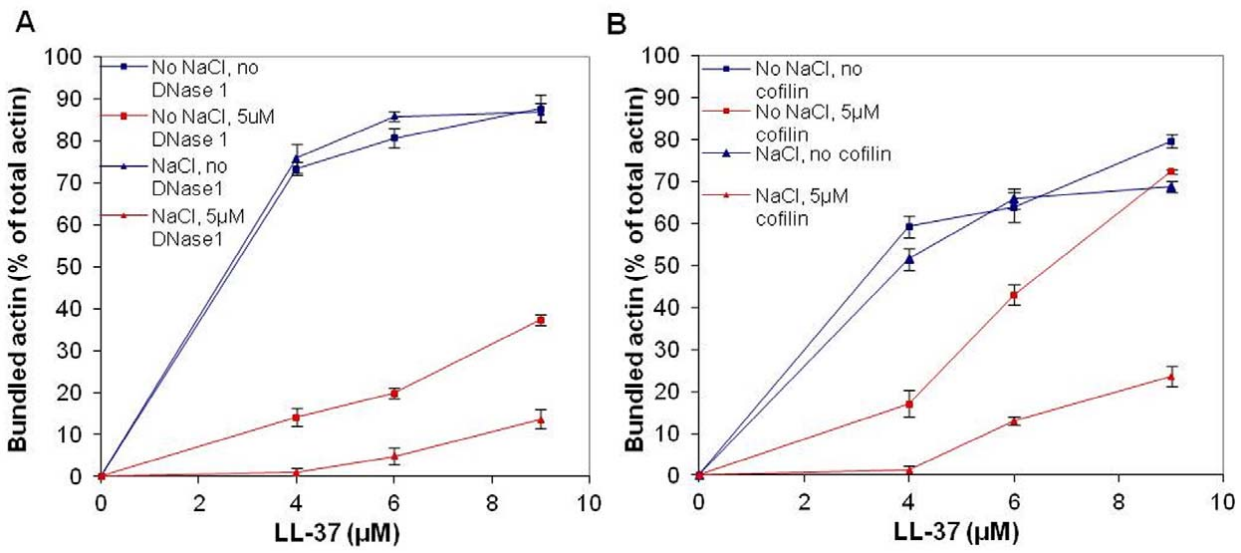
Dissociation of LL-37-induced Mg-F-actin bundles by DNase1 and cofilin at low and physiological ionic strength. 5 µM DNase1 or cofilin were added to 4 µM Mg-F-actin bundles (bundled by 4–9 µM LL-37) in the presence or absence of 100 mM NaCl. Following 30 min incubation the samples were centrifuged at 20,800×g for 8 min and the supernatants were analyzed by SDS-PAGE and densitometry. (**A**) Dissociation by DNase1. (**B**) Dissociation by cofilin. The presented data are mean and standard deviation of three independent experiments.

Cofilin, like gelsolin, is an actin-filament severing protein [39]. DNase1, depolymerizes F-actin and forms a tight complex with the actin monomer by binding to its DNase1 binding loop (D-loop) [40]. The effect of 5 µM DNase1 (Figure 7A) or cofilin (Figure 7B) on 4 µM Mg-F-actin bundled with 4–9 µM LL-37 in the presence or absence of 100 mM NaCl was examined. Both DNase1 and cofilin were found to dissociate actin bundles and their effect decreased with the increase in the LL-37 concentration. This indicates a competition for a binding site on actin between LL-37 and DNase1 or cofilin. Dissociation was more efficient in the presence of 100 mM NaCl than in low ionic strength, indicating that the increased ionic strength contributed to the disassembly of the bundles. DNase1 dissociated F-actin bundles far more efficiently than cofilin. This was especially conspicuous at low ionic strength, where cofilin had a poor dissociating effect and almost did not affect bundling at high (9 µM) LL-37 concentrations.

**Figure 8:**
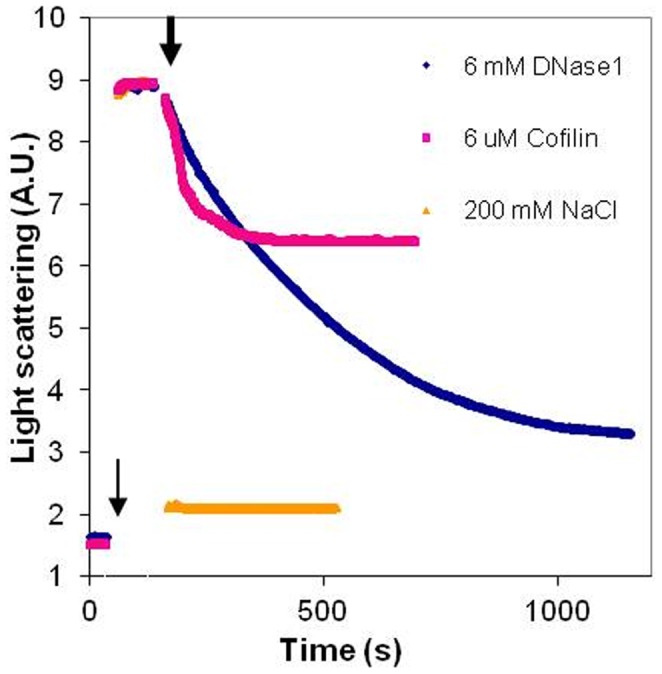
Kinetics of dissociation of MgF-actin bundles by DNase 1, cofilin and NaCl. LL-37 (9 µM) was added to 4 µM MgF-actin (thin arrow), inducing immediate bundling. This was followed by the addition of 6 µM DNase1, 6 µM cofilin, or 200 mM NaCl (thick arrow). Bundling and dissociation were followed as an increase and decrease in light scattering, respectively, measured at 450 nm. Presented data are representative of three independent experiments.

**Figure 9:**
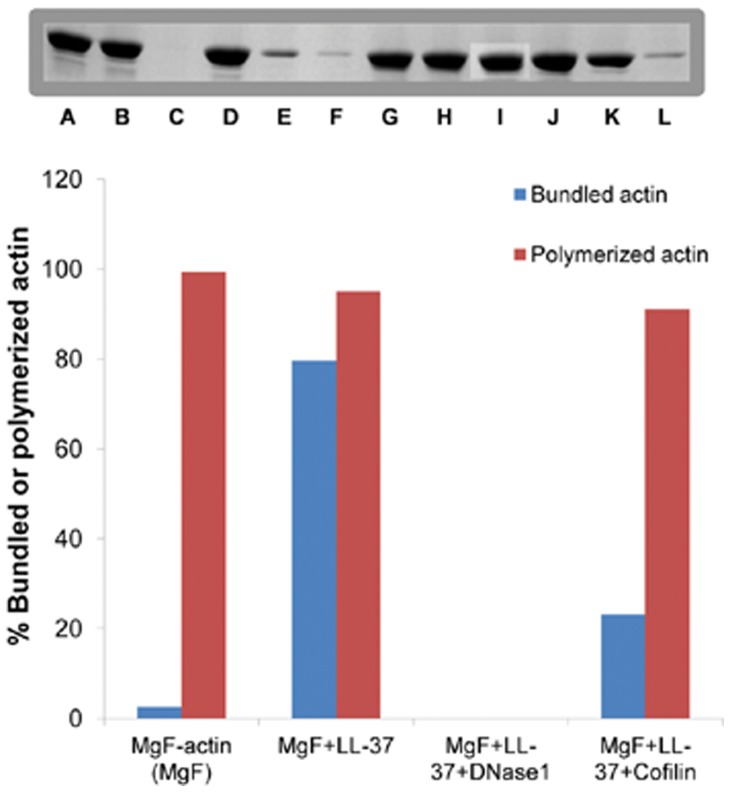
Mechanism of dissociation of LL-37-induced Mg-F-actin bundles by DNase1 and cofilin. LL-37 (9 µM) was added to 2 µM Mg-F-actin. Following a 10 minute incubation, 6 µM DNase1 or 9 µM cofilin were added. After 30 min incubation the samples were divided into two pools and centrifuged at 20,800×g or 352,271×*g* for 8 and 60 min, respectively. The supernatants were analyzed by SDS-PAGE and densitometry. Monomer (G-actin) separated from actin filaments (F-actin) by one hour 352,271×*g* centrifugation. Bundled F-actin was separated from unbundled F-actin by low speed (20,800×*g* for 8 min) centrifugation. Thus, the three actin forms (G-, unbundled F- and bundled F-actin) were separated from each other by low and high speed centrifugations. Addition of 6 µM DNase1 and 9 µM cofilin to 4 µM Mg-F-actin (bundled by 9 µM LL-37) promoted disappearance of bundles at 100% and 73%, respectively. The presented data are mean and standard deviation of three independent experiments. Gel insert shows representative actin bands: (A) MgF-actin, pre-spin; (B) MgF-actin low speed; (C) MgF-actin high speed centrifugation; (D) MgF-actin, LL-37 pre-spin; (E) MgF-actin, LL-37 low speed; (F) MgF-actin, LL-37 high speed centrifugation; (G) MgF-actin, LL-37, DNase1 pre-spin; (H) MgF-actin, LL-37, DNase1 low speed; (I) MgF-actin, LL-37, DNase1 high speed centrifugation; (J) MgF-actin, LL-37, cofilin pre-spin; (K) MgF-actin, LL-37, cofilin low speed; (L) MgF-actin, LL-37, cofilin high speed centrifugation.

**Figure 10:**
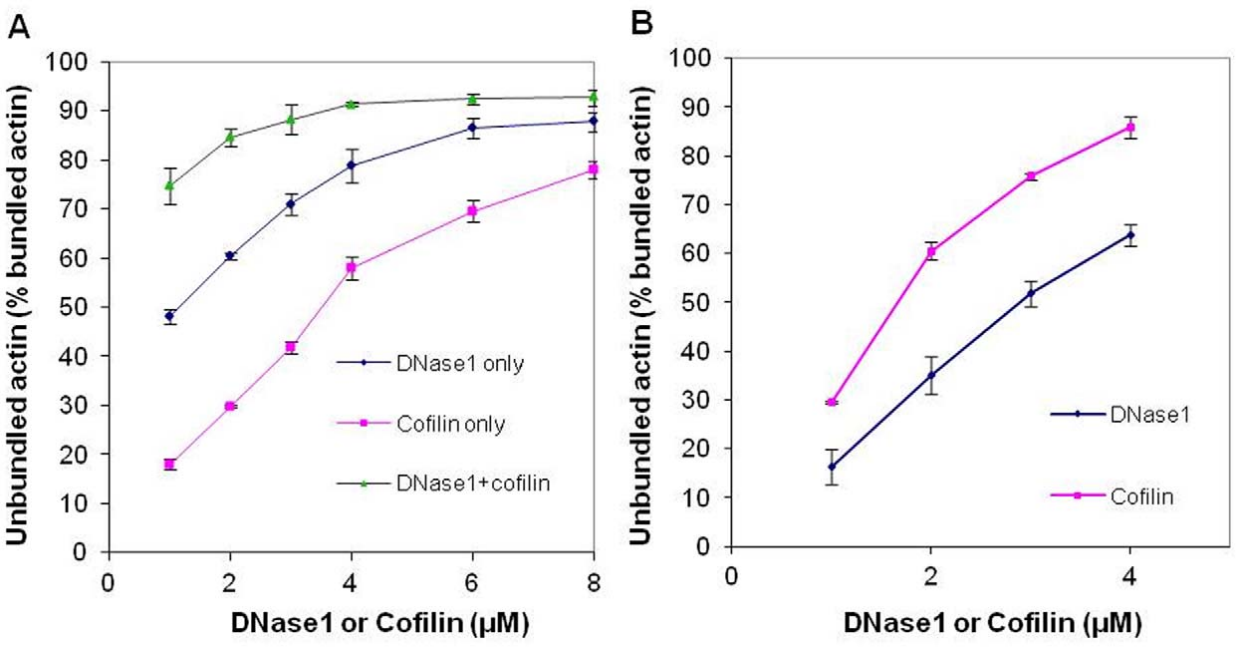
Comparison of DNase1 and cofilin dissociation of LL-37- and lysozyme- induced Mg-F-actin bundles. Mg-F-actin (4 µM) was bundled by 9 µM LL-37 or lysozyme in the presence of 100 mM NaCl. After 10 min of incubation, DNase1 or cofilin were added in increasing concentrations to bundled actin. After 30 min of incubation, the samples were centrifuged at 20,800×*g* for 8 min and the supernatants were analyzed by SDS-PAGE and densitometry. (**A**) Unbundling of LL-37-induced Mg-F-actin bundles. (**B**) Unbundling of lysozyme-induced Mg-F-actin bundles. The presented data are mean and standard deviation of three independent experiments.

The kinetics of dissociation of LL-37-induced Mg-F-actin bundles was also studied (Figure 8). In this experiment, LL-37 (9 µM) was added to MgF-actin (4 µM), which induced immediate bundling. This was followed by the addition of 6 µM DNase1, cofilin, or 200 mM NaCl. Bundling and dissociation was monitored as a change in light scattering. Dissociation was complete and very fast with 200 mM NaCl, fast but only partial with 6 µM cofilin and relatively slow but led to near complete unbundling with 6 µM DNase1. The very fast dissociation by NaCl may indicate the abolishment the interactions between the filaments. The dissociation by cofilin may be caused by severing while the slow scattering decrease induced by DNase1 can be due to depolymerization of actin filaments. These assumptions were further examined by investigating the mechanism of dissociation of LL-37-induced Mg-F-actin bundles by DNase1 and cofilin using low and high speed sedimentation (Figure 9). G-actin, which does not sediment even by high speed centrifugation, was separated from F-actin, which was sedimented by one hour centrifugation at 352,271×*g*. The bundled F-actin, unlike the unbundled one, is sedimented at low speed centrifugation (20,800×*g*, 8 min). Thus, the three actin forms (G-, unbundled F- and bundled F-actin) were separated from each other by low and high speed centrifugations. Addition of 6 µM DNase1 and 9 µM cofilin to 4 µM Mg-F-actin (bundled by 9 µM LL-37) induced bundle disappearance at 100% and 73% respectively (Figure 9). However, as indicated by the high speed centrifugation, the cofilin treated F-actin bundles remained polymerized, while the DNase1 treated one completely depolymerized (as shown by the lack of sedimentation). These results are in accordance with the well known depolymerizing effect of DNase1 through the formation of G-actin-DNase1 complex [40] and cofilin severing [41] of the actin filament. Finally, we compared the dissociation of LL-37- (Figure 10A) or lysozyme-induced (Figure 10B) actin bundles by DNase1 and cofilin. In this experiment MgF-actin (4 µM) was bundled by 9 µM LL-37 or lysozyme in the presence of 100 mM NaCl. DNase1 or cofilin were subsequently added in increasing concentrations to the bundled actin, which after 10 min incubation was separated from the unbundled actin by low speed sedimentation. The dissociation of LL-37-induced actin bundles was found to be more extensive with DNase1 than with cofilin and the unbundling effect of the two proteins was additive (Figure 10A). Contrary to the LL-37-induced bundles, the dissociation of the lysozyme-induced bundles is more extensive with cofilin than with DNase1. These findings indicate that there are structural differences between the two types of bundles. It seems that the LL-37-induced bundles are more easily depolymerized by DNase1 while for the lysozyme-induced ones the filament severing is the preferred unbundling process.

## Discussion

LL-37 is a polycationic 37-mer human host defense peptide with multiple functions that include antimicrobial, immunoregulatory and tissue repair activities [21], [42]. It polymerizes G-actin and bundles F-actin if its concentration is higher than that of actin and reacts with actin monomers or protomers also at low concentrations, which do not polymerize actin. The LL-37-induced bundles could play a role in cellular actin dynamics and may participate in the formation of cytoskeletal structures. LL-37 has been shown to have many cellular functions, including chemoattraction, cytokine release, tissue regeneration, inhibition of immunostimulation and apoptosis [21], [43]. It is assumed that the direct or indirect effects of LL-37 on actin dynamics have a significant role on antibacterial defense mechanism of lung epithelial cells [27].

LL-37 polymerizes G-actin at concentrations greater than twice than that of actin. At 4 µM LL-37, a concentration not sufficient to cause polymerization (Figure 1A), LL-37 still reacts with actin monomers, as shown by the LL-37 inhibition of MgCl_2_- and NaCl-induced actin polymerization (Figure 2A). This finding may indicate the existence of a stable LL-37-actin- monomer complex. This assumption is further supported by the acceleration of the subtilisin digestion of both CaATP-G-actin, MgATP-G-actin and MgF-actin by substoichiometric LL-37 concentrations relative to actin. Subtilisin digestion of actin is highly sensitive to subtle changes in actin structure [37]; therefore, the increased subtilisin sensitivity of actin in the presence of LL-37 indicates LL-37-induced changes in actin’s conformation. LL-37 enhanced subtilisin digestion of Mg-F-actin also in the presence of 200 mM NaCl, which dissolves LL-37-induced bundles by masking the electrostatic interactions between F-actin and LL-37. The observation that LL-37 affects actin structure even in the absence of electrostatic interactions indicates the existence of specific hydrophobic interactions between the protein and the peptide. The present investigation, which showed that F-actin bundles induced by LL-37 are significantly more resistant to increasing ionic strength compared to those formed by lysozyme and other polycations (Figure 4 and [38]), supports the existence of hydrophobic interactions between F-actin and LL-37. This was further confirmed by measuring the binding of LL-37 and scrambled LL-37 to actin by surface plasmon resonance, which showed that LL-37 strongly binds G-actin, while sLL-37 does not (Figure 6). This indicates the existence of a special actin binding sequence in LL-37, which is responsible for the hydrophobic interactions between the peptide and actin. These interactions help to stabilize the bundles formed by the polycationic peptide via neutralization of the repulsive negative charges of actin filaments.

DNase1, a G-actin binding and F-actin depolymerizing protein [40] and cofilin, an actin filament severing and depolymerization promoting protein [41], [44] and [45] were shown in the present study to disassemble LL-37- and lysozyme-induced actin bundles. DNase1 unbundles polycation-induced bundles by depolymerizing actin filaments attached to each other. This is indicated by the finding that essentially no F-actin remained in the solution after DNase1 caused disassembly of the bundles. Cofilin dissociates LL-37-induced bundles by severing filaments, as actin remained polymerized after cofilin addition (Figure 10). The DNase1-caused dissociation of LL-37-induced bundles is slower but much more efficient than the disassembly induced by cofilin. On the other hand, cofilin is more efficient than DNAse1 at dissociating lysozyme-induced bundles at physiological ionic strength. This implies possible structural differences between LL-37- and lysozyme-induced actin filament bundles. The strong dissociating effect of DNase1 on LL-37-induced bundles may indicate that DNase1 and LL-37 compete for the same site of the actin protein, specifically for the DNase1 binding (D) loop.

In cystic fibrosis, which is the most common fatal inherited disease in the western world [29], large quantities of F-actin and DNA released from lysed inflammatory cells are found in the surface airway liquid, together with polycationic antimicrobial polypeptides, such as LL-37, lysozyme, ß-defensin and lactoferrin [38]. These polycations promote the formation of actin and DNA bundles by neutralizing the repulsive negative charges of the F-actin and DNA polyanions [18], [20]. The bundles have a significant role in symptom aggravation in cystic fibrosis patients. LL-37 was previously shown to bundle actin in the sputum of cystic fibrosis patients [6]. However, in sputum of cystic fibrosis patients LL-37 concentration was found to reach ∼15 µg/ml (∼3 µM) [46] while extracellular F-actin ranged between 0.1–5 mg/ml (∼2–120 µM) [30]. This LL-37/actin ratio is seemingly lower than the superstoichiometric one found here necessary for actin bundling. It is possible that in inflamed site, temporary local LL-37 concentrations together with those of other cationic actin bundling proteins (such as lysozyme) reach sufficient concentrations to induce bundling of actin released from lysed cells.

LL-37-induced F-actin bundles have a special impact on the disease because they form at relatively low LL-37 concentrations and are stable at physiological ionic strength, unlike those induced by low micromolar concentrations of lysozyme, which are dissociated at physiological ionic strength. Another important characteristic of LL-37-induced bundles is their very efficient DNase1 induced disassembly. DNase1 dissociates DNA bundles by cleaving the DNA double helix. This activity is inhibited by actin, which is tightly bound to DNase1. Therefore, a specially constructed human recombinant DNase1, whose actin binding site is missing was developed to dissociate DNA bundles in the airways of cystic fibrosis patients [47]; however, this DNase1 is not suitable to disassemble actin bundles due to the fact that it does not react with actin. In light of the efficient dissociation of LL-37-induced actin bundles by native, non-recombinant DNase1 (used in this study), it would be worth to study the use of a mixture of non-recombinant and recombinant DNase1, the former to dissociate actin and the latter to sever DNA bundles, for cystic fibrosis treatment.

## Supporting Information

Figure S1 **LL-37 induces bundling of Mg-F-actin.** Scanning electron microscopy showing 2 µM of Mg-F-actin before (A) and after (B) incubation with 4 µM LL-37.
(TIF)

## References

[1] Rottner K, Stradal TE (2011) Actin dynamics and turnover in cell motility. Curr Opin Cell Biol 23: 569–578. 21807492 10.1016/j.ceb.2011.07.003

[2] Puius YA, Mahoney NM, Almo SC (1998) The modular structure of actin-regulatory proteins. Curr Opin Cell Biol 10: 23–34. 9484592 10.1016/s0955-0674(98)80083-5

[3] Furukawa R, Fechheimer M (1997) The structure, function, and assembly of actin filament bundles. Int Rev Cytol 175: 29–90. 9203356 10.1016/s0074-7696(08)62125-7

[4] Bartles JR (2000) Parallel actin bundles and their multiple actin-bundling proteins. Curr Opin Cell Biol 12: 72–78. 10679353 10.1016/s0955-0674(99)00059-9 PMC2853926

[5] Tang JX, Janmey PA (1996) The polyelectrolyte nature of F-actin and the mechanism of actin bundle formation. J Biol Chem 271: 8556–8563. 8621482 10.1074/jbc.271.15.8556

[6] Weiner DJ, Bucki R, Janmey PA (2003) The antimicrobial activity of the cathelicidin LL37 is inhibited by F-actin bundles and restored by gelsolin. Am J Respir Cell Mol Biol 28: 738–745. 12600826 10.1165/rcmb.2002-0191OC

[7] Sanders LK, Xian W, Guaqueta C, Strohman MJ, Vrasich CR, et al (2007) Control of electrostatic interactions between F-actin and genetically modified lysozyme in aqueous media. Proc Natl Acad Sci U S A 104: 15994–15999. 17911256 10.1073/pnas.0705898104 PMC2042150

[8] Bubb MR, Lenox RH, Edison AS (1999) Phosphorylation-dependent conformational changes induce a switch in the actin-binding function of MARCKS. J Biol Chem 274: 36472–36478. 10593944 10.1074/jbc.274.51.36472

[9] Yarmola EG, Edison AS, Lenox RH, Bubb MR (2001) Actin filament cross-linking by MARCKS: characterization of two actin-binding sites within the phosphorylation site domain. J Biol Chem 276: 22351–22358. 11294839 10.1074/jbc.M101457200

[10] Harbeck B, Huttelmaier S, Schluter K, Jockusch BM, Illenberger S (2000) Phosphorylation of the vasodilator-stimulated phosphoprotein regulates its interaction with actin. J Biol Chem 275: 30817–30825. 10882740 10.1074/jbc.M005066200

[11] Beall B, Chalovich JM (2001) Fesselin, a synaptopodin-like protein, stimulates actin nucleation and polymerization. Biochemistry 40: 14252–14259. 11714279 10.1021/bi011806u PMC1289264

[12] Schroeter M, Chalovich JM (2004) Ca2+-calmodulin regulates fesselin-induced actin polymerization. Biochemistry 43: 13875–13882. 15504050 10.1021/bi0487490

[13] Tang JX, Szymanski PT, Janmey PA, Tao T (1997) Electrostatic effects of smooth muscle calponin on actin assembly. Eur J Biochem 247: 432–440. 9249057 10.1111/j.1432-1033.1997.00432.x

[14] Winder SJ, Walsh MP (1993) Calponin: thin filament-linked regulation of smooth muscle contraction. Cell Signal 5: 677–686. 8130072 10.1016/0898-6568(93)90029-l

[15] Oriol-Audit C (1978) Polyamine-induced actin polymerization. Eur J Biochem 87: 371–376. 149658 10.1111/j.1432-1033.1978.tb12386.x

[16] Sowa GZ, Cannell DS, Liu AJ, Reisler E (2006) Polyamine-induced bundling of F-actin. J Phys Chem B 110: 22279–22284. 17078670 10.1021/jp063371w

[17] Brown SS, Spudich JA (1979) Nucleation of polar actin filament assembly by a positively charged surface. J Cell Biol 80: 499–504. 572366 10.1083/jcb.80.2.499 PMC2110329

[18] Tang JX, Ito T, Tao T, Traub P, Janmey PA (1997) Opposite effects of electrostatics and steric exclusion on bundle formation by F-actin and other filamentous polyelectrolytes. Biochemistry 36: 12600–12607. 9376366 10.1021/bi9711386

[19] Grintsevich EE, Phillips M, Pavlov D, Phan M, Reisler E, et al (2010) Antiparallel dimer and actin assembly. Biochemistry 49: 3919–3927. 20361759 10.1021/bi1002663 PMC3133779

[20] Muhlrad A, Grintsevich EE, Reisler E (2011) Polycation induced actin bundles. Biophys Chem 155: 45–51. 21411219 10.1016/j.bpc.2011.02.008 PMC3086713

[21] Bucki R, Leszczynska K, Namiot A, Sokolowski W (2010) Cathelicidin LL-37: a multitask antimicrobial peptide. Arch Immunol Ther Exp (Warsz) 58: 15–25. 20049649 10.1007/s00005-009-0057-2

[22] De Y, Chen Q, Schmidt AP, Anderson GM, Wang JM, et al (2000) LL-37, the neutrophil granule- and epithelial cell-derived cathelicidin, utilizes formyl peptide receptor-like 1 (FPRL1) as a receptor to chemoattract human peripheral blood neutrophils, monocytes, and T cells. J Exp Med 192: 1069–1074. 11015447 10.1084/jem.192.7.1069 PMC2193321

[23] Elssner A, Duncan M, Gavrilin M, Wewers MD (2004) A novel P2X7 receptor activator, the human cathelicidin-derived peptide LL37, induces IL-1 beta processing and release. J Immunol 172: 4987–4994. 15067080 10.4049/jimmunol.172.8.4987

[24] Carretero M, Escamez MJ, Garcia M, Duarte B, Holguin A, et al (2008) In vitro and in vivo wound healing-promoting activities of human cathelicidin LL-37. J Invest Dermatol 128: 223–236. 17805349 10.1038/sj.jid.5701043

[25] Rosenfeld Y, Papo N, Shai Y (2006) Endotoxin (lipopolysaccharide) neutralization by innate immunity host-defense peptides. Peptide properties and plausible modes of action. J Biol Chem 281: 1636–1643. 16293630 10.1074/jbc.M504327200

[26] Nagaoka I, Tamura H, Hirata M (2006) An antimicrobial cathelicidin peptide, human CAP18/LL-37, suppresses neutrophil apoptosis via the activation of formyl-peptide receptor-like 1 and P2X7. J Immunol 176: 3044–3052. 16493063 10.4049/jimmunol.176.5.3044

[27] Lau YE, Rozek A, Scott MG, Goosney DL, Davidson DJ, et al (2005) Interaction and cellular localization of the human host defense peptide LL-37 with lung epithelial cells. Infect Immun 73: 583–591. 15618198 10.1128/IAI.73.1.583-591.2005 PMC538997

[28] Byfield FJ, Kowalski M, Cruz K, Leszczynska K, Namiot A, et al (2011) Cathelicidin LL-37 increases lung epithelial cell stiffness, decreases transepithelial permeability, and prevents epithelial invasion by *Pseudomonas aeruginosa* . J Immunol 187: 6402–6409. 22095714 10.4049/jimmunol.1102185

[29] Welsh MJ, Smith AE (1995) Cystic fibrosis. Sci Am 273: 52–59. 8525348 10.1038/scientificamerican1295-52

[30] Vasconcellos CA, Allen PG, Wohl ME, Drazen JM, Janmey PA, et al (1994) Reduction in viscosity of cystic fibrosis sputum in vitro by gelsolin. Science 263: 969–971. 8310295 10.1126/science.8310295

[31] Spudich JA, Watt S (1971) The regulation of rabbit skeletal muscle contraction. I. Biochemical studies of the interaction of the tropomyosin-troponin complex with actin and the proteolytic fragments of myosin. J Biol Chem 246: 4866–4871. 4254541

[32] Kouyama T, Mihashi K (1981) Fluorimetry study of N-(1-pyrenyl)iodoacetamide-labelled F-actin. Local structural change of actin protomer both on polymerization and on binding of heavy meromyosin. Eur J Biochem 114: 33–38. 7011802

[33] Bradford MM (1976) A rapid and sensitive method for the quantitation of microgram quantities of protein utilizing the principle of protein-dye binding. Anal Biochem 72: 248–254. 942051 10.1016/0003-2697(76)90527-3

[34] Canfield RE (1963) The Amino Acid Sequence of Egg White Lysozyme. J Biol Chem 238: 2698–2707. 14063294

[35] Schwyter D, Phillips M, Reisler E (1989) Subtilisin-cleaved actin: polymerization and interaction with myosin subfragment 1. Biochemistry 28: 5889–5895. 2673352 10.1021/bi00440a027

[36] Strzelecka-Golaszewska H, Moraczewska J, Khaitlina SY, Mossakowska M (1993) Localization of the tightly bound divalent-cation-dependent and nucleotide-dependent conformation changes in G-actin using limited proteolytic digestion. Eur J Biochem 211: 731–742. 8436131 10.1111/j.1432-1033.1993.tb17603.x

[37] Muhlrad A, Kudryashov D, Michael Peyser Y, Bobkov AA, Almo SC, et al (2004) Cofilin induced conformational changes in F-actin expose subdomain 2 to proteolysis. J Mol Biol 342: 1559–1567. 15364581 10.1016/j.jmb.2004.08.010

[38] Bucki R, Byfield FJ, Janmey PA (2007) Release of the antimicrobial peptide LL-37 from DNA/F-actin bundles in cystic fibrosis sputum. Eur Respir J 29: 624–632. 17215317 10.1183/09031936.00080806

[39] Bamburg JR (1999) Proteins of the ADF/cofilin family: essential regulators of actin dynamics. Annu Rev Cell Dev Biol 15: 185–230. 10611961 10.1146/annurev.cellbio.15.1.185

[40] Kabsch W, Mannherz HG, Suck D, Pai EF, Holmes KC (1990) Atomic structure of the actin:DNase I complex. Nature 347: 37–44. 2395459 10.1038/347037a0

[41] Pavlov D, Muhlrad A, Cooper J, Wear M, Reisler E (2007) Actin filament severing by cofilin. J Mol Biol 365: 1350–1358. 17134718 10.1016/j.jmb.2006.10.102 PMC2572264

[42] Nijnik A, Hancock RE (2009) The roles of cathelicidin LL-37 in immune defences and novel clinical applications. Curr Opin Hematol 16: 41–47. 19068548 10.1097/moh.0b013e32831ac517

[43] Bowdish DM, Davidson DJ, Hancock RE (2006) Immunomodulatory properties of defensins and cathelicidins. Curr Top Microbiol Immunol 306: 27–66. 16909917 10.1007/3-540-29916-5_2 PMC7121507

[44] Andrianantoandro E, Pollard TD (2006) Mechanism of actin filament turnover by severing and nucleation at different concentrations of ADF/cofilin. Mol Cell 24: 13–23. 17018289 10.1016/j.molcel.2006.08.006

[45] Pavlov D, Muhlrad A, Cooper J, Wear M, Reisler E (2006) Severing of F-actin by yeast cofilin is pH-independent. Cell Motil Cytoskeleton 63: 533–542. 16847879 10.1002/cm.20142 PMC2583072

[46] Chen CI, Schaller-Bals S, Paul KP, Wahn U, Bals R (2004) Beta-defensins and LL-37 in bronchoalveolar lavage fluid of patients with cystic fibrosis. J Cyst Fibros 3: 45–50. 15463886 10.1016/j.jcf.2003.12.008

[47] Ulmer JS, Herzka A, Toy KJ, Baker DL, Dodge AH, et al (1996) Engineering actin-resistant human DNase I for treatment of cystic fibrosis. Proc Natl Acad Sci U S A 93: 8225–8229. 8710851 10.1073/pnas.93.16.8225 PMC38651

