# Supplementary figures and images for: LL-37 Induces Polymerization and Bundling of Actin and Affects Actin Structure
Source: PLoS One. 2012 Nov 26;7(11):e50078. doi: 10.1371/journal.pone.0050078 (PMC3506534; doi:10.1371/journal.pone.0050078)

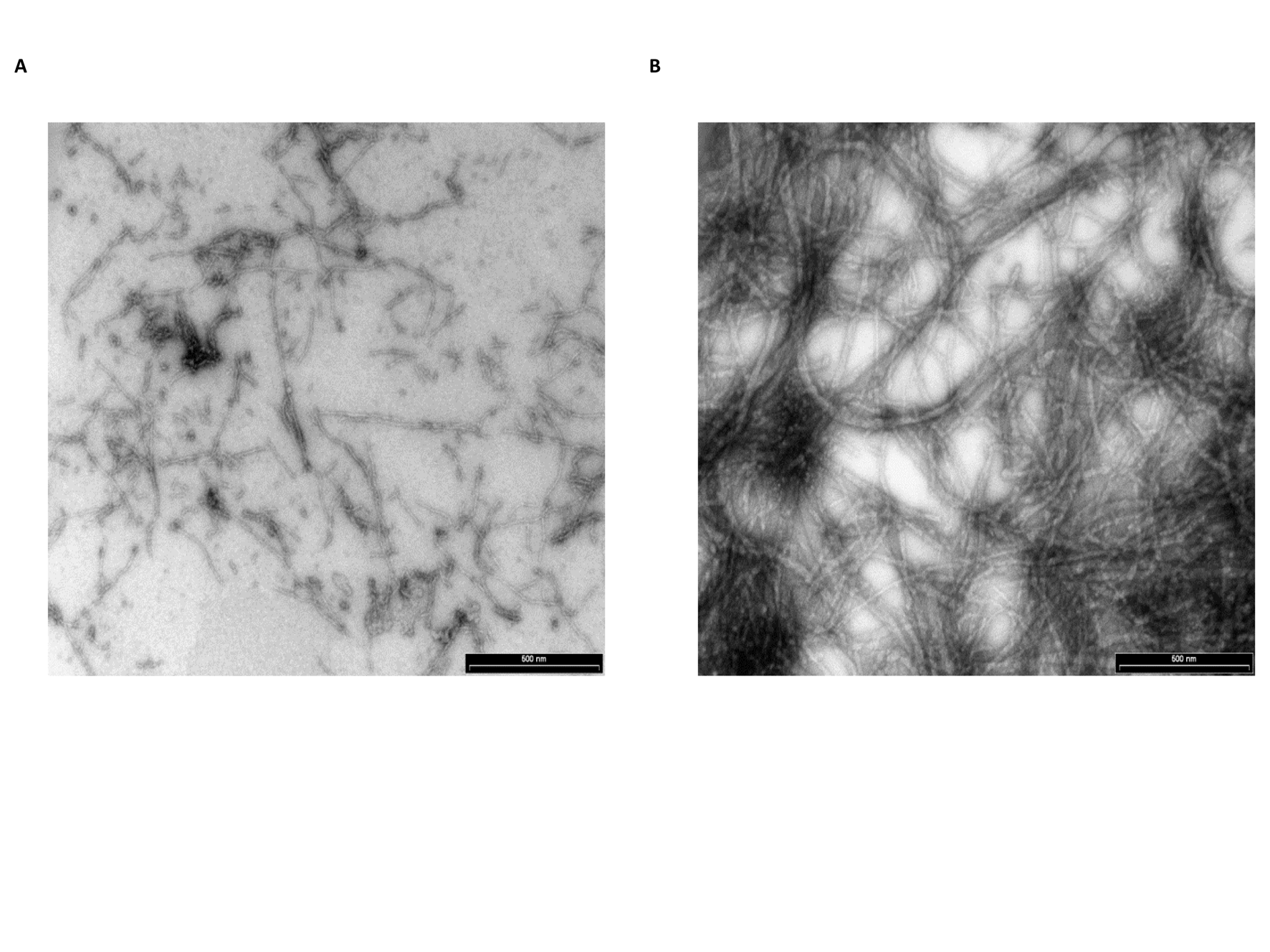

Supplement: Figure S1 — LL-37 induces bundling of Mg-F-actin. Scanning electron microscopy showing 2 µM of Mg-F-actin before (A) and after (B) incubation with 4 µM LL-37. (TIF) [file pone.0050078.s001.tif]
